# In vivo imaging of endogenous enzyme activities using luminescent 1,2-dioxetane compounds

**DOI:** 10.1186/s12929-015-0155-x

**Published:** 2015-06-24

**Authors:** Jen-Chieh Tseng, Andrew L. Kung

**Affiliations:** Lurie Family Imaging Center, Dana-Farber Cancer Institute, 450 Brookline Avenue, Boston, MA 02215 USA; Columbia University Medical Center, 3959 Broadway, New York, NY 10032 USA; Current address: PerkinElmer Inc, 68 Elm Street, Hopkinton, MA 01748 USA

**Keywords:** *In vivo* imaging, Luminescence, Fluorescence, 1,2-dioxetane, CIEEL energy transfer

## Abstract

**Background:**

Here we present a non-invasive imaging method for visualizing endogenous enzyme activities in living animals. This optical imaging method is based on an energy transfer principle termed chemically initiated electron exchange luminescence (CIEEL). The light energy is provided by enzymatic activation of metastable 1,2-dioxetane substrates, whose protective groups are removed by hydrolytic enzymes such as β-galactosidase and alkaline phosphatase. In the presence of a nearby fluorescent recipient, the chemical energy within the activated substrate is then transferred via formation of a charge-transfer complex with the fluorophore, a mechanism closely related to glow stick chemistry.

**Results:**

Efficient CIEEL energy transfer requires close proximity between the trigger enzyme and the fluorescent recipient. Using cells stained with fluorescent dialkylcarbocyanines as the energy recipients, we demonstrated CIEEL imaging of cellular β-galactosidase or alkaline phosphatase activity. In living animals, we used a similar approach to non-invasively image alkaline phosphatase activity in the peritoneal cavity.

**Conclusions:**

In this report, we provide proof-of-concept for CIEEL imaging of *in vivo* enzymatic activity. In addition, we demonstrate the use of CIEEL energy transfer for visualizing elevated alkaline phosphatase activity associated with tissue inflammation in living animals.

## Background

Non-invasive optical imaging of small laboratory animals has been an important tool for biomedical research in pre-clinical settings. Light signals are typically generated within the body using fluorescent or luminescent methods. Fluorescence imaging methods use external excitation as the energy source for signal generation [1]. In order to achieve deep tissue penetration, several near-infrared (NIR) fluorescent molecules and nanoparticles have been developed for *in vivo* imaging applications. Many of these probes have been conjugated with targeting moieties, such as antibodies and protein ligands, for specific targeting and delivery [1]. In addition, since many fluorescent agents change their optical properties in response to bio-physiological changes, such as pH, membrane potential and oxidative stress, fluorescence imaging is versatile and allows for development of a broad range of probes as reporters for different biological applications [2]. Nevertheless, fluorescent methods in general have low signal-to-noise (S/N) ratios due to potential interference of excessive excitation photons. Furthermore, successful fluorescence imaging relies on the pharmacokinetic properties of the fluorescent agents to establish contrast in the target tissues [3]. On the other hand, luminescence imaging uses chemical substrates as the energy sources [4, 5]. Without the interference of external light, luminescence imaging is in general more sensitive and has better S/N ratios than fluorescent methods [6]. For example, bioluminescence imaging (BLI) has been widely used for *in vivo* imaging in small laboratory animals [4]. BLI generates light signals using the luciferases found in light-producing organisms. Luciferases are unique enzymes capable of light production by catalyzing oxidation of their specific luciferin substrates. In particular, firefly luciferase is commonly used for BLI and its expression can be ectopically introduced into mammalian cells for imaging purposes. In the presence of its specific substrate D-luciferin, BLI is a measure of cell number and migration in living animals [4]. However, compared with fluorescence imaging, BLI is less flexible since it requires genetic modifications of the target cells or tissues for luciferase expression and depends on the use of compatible luciferin substrates for light production [4].

We have previously demonstrated a hybrid optical imaging method that combines the advantages of both luminescence and fluorescence imaging [7]. The method is based on an energy transfer mechanism termed chemically initiated electron exchange luminescence (CIEEL) [8]. Using a chemiluminescent compound MCLA as the energy source and a mitochondria-targeting JC-1 fluorescent dye as the chemical energy recipient, we were able to visualize endogenous reactive oxygen species (ROS) production in living animals [7]. MCLA, or 6-(4-methoxyphenyl)-2-methyl-3,7-dihydroimidazo[1,2-*a*]pyrazin-3-one hydrochloride, is a *Cypridina* luciferin analog that can be activated by ROS to form a high-energy 1,2-dioxetane derivative [9, 10]. Importantly, the chemical energy stored within the high-energy intermediate can be transferred via formation of a charge-exchange complex with the nearby fluorescent recipient, which then emits light according to the fluorophore’s emission property [7]. This energy transfer mechanism is very similar to the glow stick chemistry where the peroxalate bis-2,4,6-(trichlorophenyl)oxalate (TCPO) is used as the energy source to generate high-energy 1,2-dioxetane derivatives [11]. Since mammalian mitochondria constantly produce ROS as byproducts of oxidative phosphorylation, targeting the organelle with a mitochondria-targeting JC-1 fluorescent dye enables visualization of endogenous ROS production *in vivo* [7]. Beside mitochondria, phagocytes produce high levels of ROS in the phagosomes during active phagocytosis of invading bacteria [12], a phenomenon that can also be imaged by CIEEL energy transfer using MCLA and a phagosome-targeting fluorescent dye [7].

CIEEL energy transfer mechanism is different from resonance-based energy transfer mechanisms, such as bioluminescence resonance energy transfer (BRET) and chemiluminescence resonance energy transfer (CRET) [13, 14]. Both BRET and CRET are based on a mechanism similar to the Förster resonance energy transfer (FRET), in which efficient energy resonance requires spectral overlap between the donor’s emission spectrum and the recipient’s absorption spectrum. In other words, both donor and recipient molecules have to be “in sync” for efficient energy transfer. In contrast, CIEEL energy transfer is a Dexter mechanism in which electron exchange occurs between the energy donor 1,2-dioxetane and the fluorescent recipient [8]. This alternative mechanism implies direct intermolecular collision between the donor and recipient molecules, and energy transfer occurs without spectral requirement for the donor and recipient. As a result, the CIEEL imaging technique is capable of generating a variety of emission colors by using a single high-energy substrate. Glow sticks are good examples of CIEEL energy transfer luminescence, which are capable of generating a variety of colors using a single peroxalate TCPO compound as the energy source [11, 15]. The color of a glow stick is solely determined by the recipient fluorescent dye of choice. Thus, CIEEL is a hybrid imaging technique with the advantages of luminescence and fluorescence imaging. Without the use of an external light source, CIEEL has lower background noise in comparison with the conventional fluorescence imaging methods. Meanwhile, CIEEL imaging is more flexible than bioluminescence imaging as it can adapt to a greater variety of fluorescent recipients for different emission colors.

**Fig. 1:**
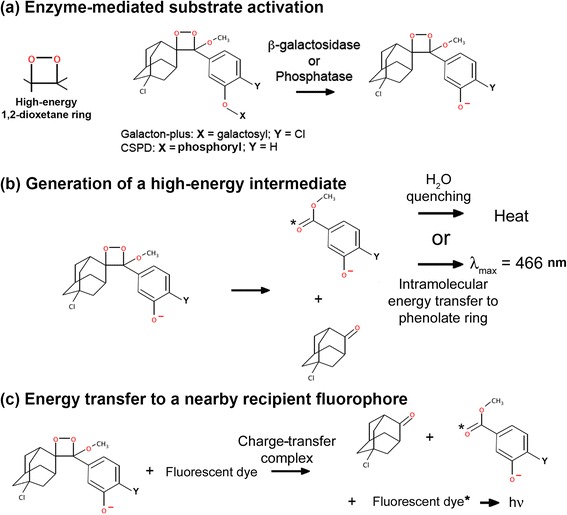
CIEEL energy transfer for imaging enzyme activities. **a** Chemical structures of 1,2-dioxetane chemiluminescent substrates and their activation by hydrolytic enzymes. Galacton-plus is a substrate for β-galatosidase, and CSPD is an alkaline phosphatase substrate. The hydrolytic activity of β-galatosidase or alkaline phosphatase removes the protective group X and results in substrate activation. **b** In the absence of a recipient fluorescent dye, the activated substrate self-decomposes and results in blue chemiluminescence emission or heat release due to water quenching. **c** In the presence of a fluorescent recipient, the activated substrate can directly transfer its chemical energy via formation of a charge-transfer complex with the recipient fluorophore

In addition to peroxalate-based compounds, several 1,2-dioxetane chemiluminescent substrates have been widely used for biochemical detection of enzyme activities *in vitro* such as β-galactosidase (Galacton-plus®, Fig. 1a) and alkaline phosphatase (CSPD®, Fig. 1a) [16]. Upon removal of the protective galactosyl or phosphoryl group, the negative electrical charge on the phenolate group triggers an electron transfer event that in turn induces decomposition of the high-energy 1,2-dioxetane ring (Fig. 1a) [16]. The decomposition would generate several high-energy intermediates in excited states. One possibility is generating an excited methyl-benzene carboxylate moiety. The fates of the excited intermediates would largely depend upon the surrounding environments. In an aqueous solution, the majority of excited intermediates are quenched by surrounding water molecules and results in heat release (Fig. 1b) [16]. It is unavoidable to have heat release during CIEEL imaging due to the ubiquitous presence of water in biological systems. Nevertheless, the extent of water quenching should not vary considerably among different animal subjects as water activity tends to be well controlled in tissues. Reducing water activity by adding macromolecule enhancers, such as polymer micelles, protects them from water quenching and thus promotes energy released through the phenolate ring as blue chemiluminescence (466 nm, Fig. 1b). However, blue light is not suitable for *in vivo* imaging due to its strong absorbance by hemoglobin. Redder emission can be achieved by coupling the activated substrate with a nearby recipient fluorophore. The activated substrate can directly transfer its chemical energy to the fluorescent dye via intermolecular CIEEL energy transfer (Fig. 1c). Of note, it is possible to use a near-infrared fluorescent probe as the energy recipient in order to further enhance tissue penetration and signal strength. In this study, we examine the use of 1,2-dioxetane substrates for visualizing enzyme activities in biological systems. In particular, we demonstrate the use of this novel concept for imaging increased alkaline phosphatase activity associated with tissue inflammation.

## Methods

### Cell lines

The human colon cancer HCT116 cells were cultured in DMEM supplied with 10 % FCS. The cells were stably transfected with a plasmid pBMN-Z-IN (Addgene Inc., Cambridge MA) carrying a LacZ-IRES-Neo cassette for bacterial β-galactosidase expression. The resulting cells, HCT116/LacZ were maintained under selection with G418 at 1 mg/mL. The human multiple myeloma MM1S cells were maintained in RPMI 1640 medium with 10 % FCS.

### Energy transfer imaging of β-galactosidase activity

For energy transfer imaging of biotin-streptavidin conjugates, each well contained 300 μL total reaction in PBS including: 1 μg biotinylated β-galacotidase (biotin/protein = 2.91, 430 units/mg protein, from E. coli, Sigma-Aldrich, St. Louis MO), 1 μL of Galacton-Plus concentrate (Applied Biosystems, Foster City CA), 0–160 pmol of ovalbumin-Alexa Fluor 647 conjugate (Invitrogen, Carlsbad CA) or 0–160 pmol of streptavidin-Alexa Fluor 647 conjugate (Invitrogen, Carlsbad CA). Luminescence was imaged using the IVIS Spectrum System (PerkinElmer, Waltham MA) without any emission filter (open) for total luminescence output, or individually with emission filters at 500 or 680 nm. The 500 nm luminescence indicated self-decompostion chemiluminescence levels and the 680 nm luminescence indicated Alexa-Fluor 647 CIEEL energy transfer signal. Fluorescent images were also acquired with an excitation filter at 640 nm and an emission filter at 680 nm to determine the input level of Alexa Fluor in each well.

For energy transfer imaging of β-galactosidase activity in cells, we stained the plasma membrane of HCT116/LacZ cells with dialkylcarboncyanine DiD, DiI, or DiR (Invitrogen, Carlsbad CA). Pellets of 1x10^7^ HCT116/LacZ cells were resuspended in 2.5 mL of staining solution and incubate at 37 °C for 15 min. For single-stained cells, the staining solution contained 0.2 μM of dye in HBSS. For double-stained cells, the staining solution contained 0.1 μM of each dye in HBSS. After incubation, cells were washed with fresh HBSS and resuspended in 1 mL of HBSS. To visualize dialkylcarboncyanine energy transfer, 100 μL of cell suspension was transferred to a black 96-well luminescent plate, and 100 μL of HBSS containing 2 μL of Galacton-Plus concentrate was added into each well. Images were first acquired with an open filter and then acquired from 500 nm to 840 nm at 20 nm intervals (1 min, binning =16, f1 for each emission filter). Spectral unmixing was performed using the Living Image software (PerkinElmer, Waltham MA). A composite image was generated where the green pseudocolor indicated the DiD CIEEL signal and the red pseudocolor indicated the DiR CIEEL signal.

MM1S cells were stained with HBSS containing 1 μM of DiR. After incubation at 37 °C for 15 min, cells were pelleted and resuspended in fresh HBSS. To generate DiR energy transfer, 100 μL of DiR-stained MM1S-LucNeo cell suspension (10 million) was added into 100 μL of HBSS containing 2 μL of CSPD alkaline phosphatase substrate (Applied Biosystems) in a black 96-well plate. Non-stained MM1S-LucNeo cells were included as negative controls. Cell luminescence emission was scanned from 500 to 840 nm.

### Energy transfer imaging of endogenous phosphatase activity

All animal procedures and imaging protocols are performed under DFCI institutional IACUC guidelines. Splenocytes were harvested from NCr nude mice (Charles River Laboratories, Wilmington MA) and were treated with RBC lysis buffer (Qiagen, Valencia CA). The cells were then washed with HBSS prior to a 24 h culture period in RPMI 1640 containing 10 % FCS and 5 mg/mL of lipopolysaccharide (LPS, from *Samonella enterica*, Sigma Aldrich). The cells were collected and then stained with HBSS containing 1 μM of DiR. To generate light signal, the DiR-stained splenocytes suspension (100 μL, 1x10^7^) was transferred into 100 μL of HBSS containing 2 μL of CSPD alkaline phosphatase substrate (Applied Biosystems) on a black 96-well plate. Untreated splenocytes cells were used as negative controls. For *in vivo* imaging, NCr nude mice received i.p. injection of 10 mg/kg LPS (Sigma Aldrich) in normal saline. In some animals, dexamethasone sodium phosphate (10 mg/kg, American Regent Inc., Shirley NY) was given 1 h prior to LPS challenge. 24 h later, the animal received an i.p. injection of 400 μL solution containing 50 % CSPD, 50 % reaction diluent, and 0.25 mg/mL DiR, prior to imaging. Cell and animal luminescence were scanned from 520 to 800 nm in 40 nm steps. 

## Results

### Enzyme-mediated energy transfer imaging using a 1,2-dioxetane substrate

**Fig. 2:**
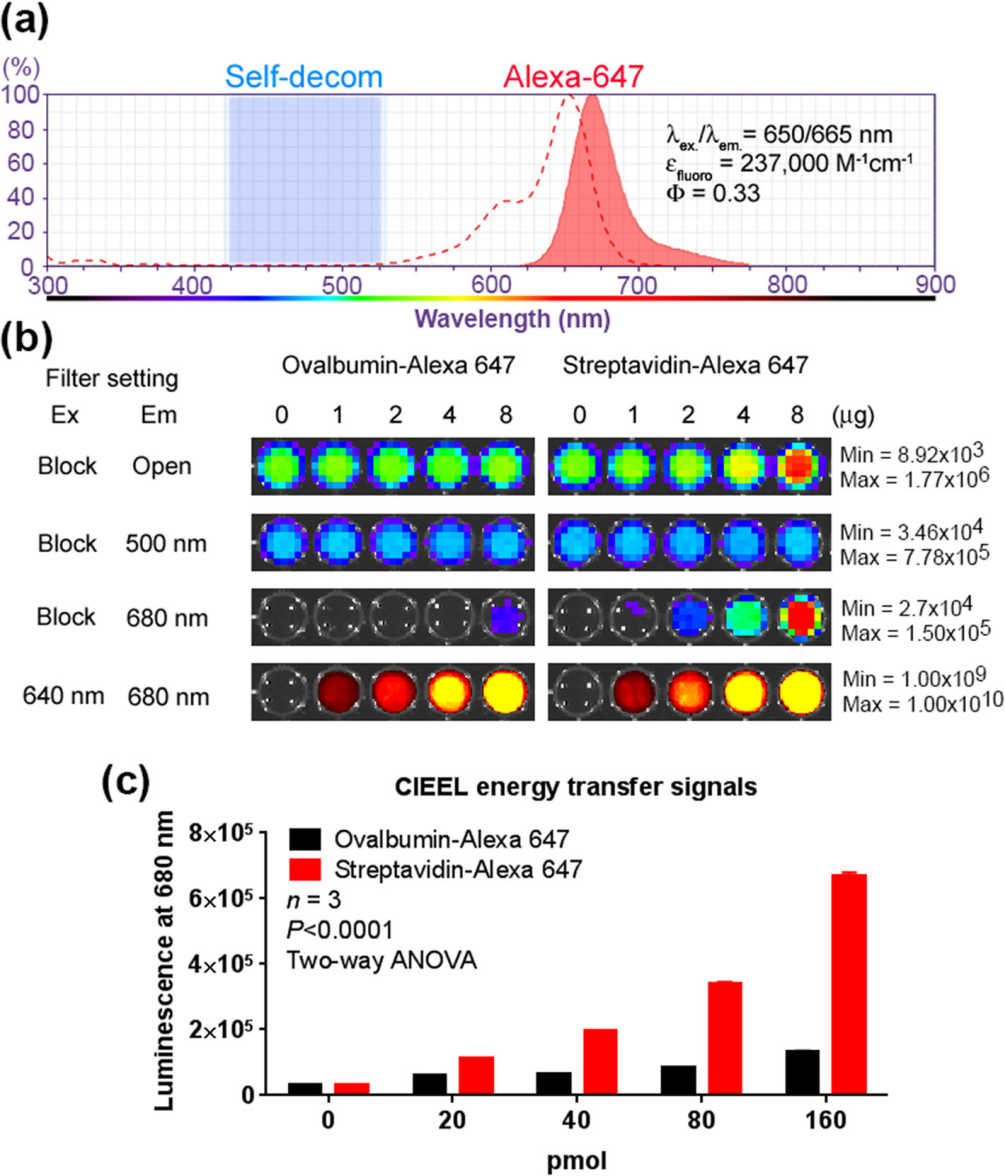
CIEEL energy transfer requires close proximity of the energy source and the recipient fluorophore. **a** The absorption/emission spectra of Alexa Fluor 647 (red), and the chemiluminescence emission range of Galacton-plus (blue shaded region). **b** Each well contained equal amounts of biotinylated β-galactosidase for Galacton-plus activation. Increasing levels of streptavidin- or ovalbumin-conjugated Alexa Fluor 647 (0–160 pmol) were added into the wells. The total luminescence output of each well was first visualized without an emission filter (open). The plate was then imaged at 500 nm (self-decomposition) or 680 nm (CIEEL energy transfer). The levels of Alexa Fluor 647 were determined by a fluorescence imaging using an Ex 640/Em 680 nm filter set. **c** Quantitative representation of the CIEEL energy transfer at 680 nm. Error bar = SEM

Galacton-plus is a 1,2-dioxetane substrate that has been commonly used in a variety of chemiluminescent assays for detecting β-galactosidase activity in aqueous samples. In order to demonstrate intermolecular CIEEL energy transfer *in vitro*, we took advantage of biotin-streptavidin interaction to bring together both the bacterial β-galactosidase enzyme and the Alexa Fluor 647 fluorophore (Fig. 2). The biotinylated β-galactosidase served as the trigger enzyme and the streptavidin-conjugated Alexa Fluor 647 functioned as the energy recipient. We reasoned that the biotin-conjugated β-galactosidase would catalyze the 1,2-dioxetane Galacton-plus substrate to release high-energy intermediates. As the biotin-streptavidin interaction brought both moieties in close proximity, the streptavidin-conjugated Alexa Fluor 647 could capture the chemical energy within the intermediates and emit a red luminescence. It is known that resonance energy transfer requires sufficient spectral overlap between the donor’s emission and the recipient's excitation spectra. However, since the Galacton-plus is a blue chemiluminescence substrate, there is virtually no spectral overlap between Galacton-plus’ blue emission and Alexa Fluor’s red excitation spectra (Fig. 2a).

Increasing levels of streptavidin-Alexa Fluor 647 fluorescent conjugates were added into wells that contain the biotinylated β-galactosidase and the Galacton-plus substrate (Fig. 2b). After adding Glacton-plus, we used two emission filters for luminescence imaging. The blue 500 nm emission filter was used to determine the conventional blue chemiluminescence of Galacton-plus, and the red 680 nm filter was used to determine the levels of energy transfer to Alexa Fluor 647. Ovalbumin-Alexa Fluor 647 conjugate that cannot bind to biotin was used as a control. Since equal amounts of β-galactosidase were used in all wells, the blue luminescence corresponding to self-decomposition (500 nm) was not affected by increasing levels of either fluorescent conjugates. Nevertheless, we observed higher levels of total luminescence (open filter) in wells containing fluorescent streptavidin conjugates (Fig. 2b). In contrast, the ovalbumin fluorescent conjugate control did not substantially enhance the total emission output. Also, we observed that the specific binding between streptavidin-Alexa Fluor 647 and the biotinylated β-galactosidase significantly enhanced the CIEEL energy transfer at 680 nm (Fig. 2c). Interestingly, this finding suggests that, after enzyme-mediated substrate activation, a portion of the chemical energy results in wasteful heat release due to water quenching. However, in the presence of nearby Alexa Fluor 647, the lost chemical energy can be recaptured and converted it to CIEEL energy transfer luminescence (680 nm), which in turn increases the total luminescence output. Since there is minimal spectral overlap between Galacton-plus emission and Alexa Fluor 647 absorption, the observed red 680 nm luminescence is not likely due to a resonance-based energy transfer between the activated substrate and the dye, but rather mediated by the charge-transfer complex mechanism that is similar to the glow stick chemistry.

### CIEEL energy transfer imaging of enzyme activities in cells

**Fig. 3:**
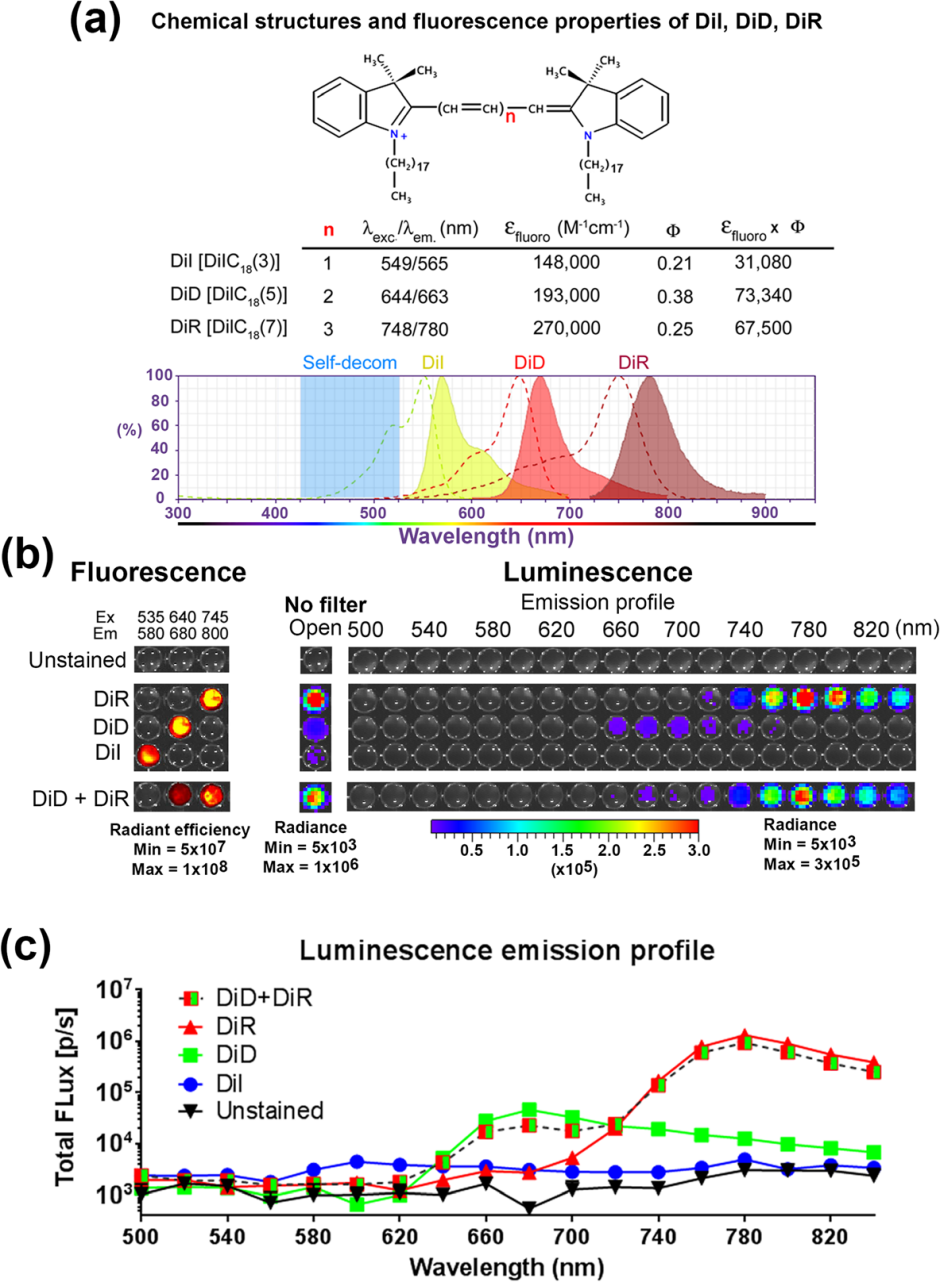
CIEEL energy transfer imaging of enzyme activities in cells. **a** Chemical structures and optical properties of three membrane-targeting fluorescent dialkylcarboncyanines, DiI, DiD, and DiR. The blue shaded region indicates the self-decomposition light emission of Galacton-plus. **b** Human HCT116/LacZ colon cancer cells were single-stained with DiI, DiD, DIR or double-stained with both DiD and DiR prior to CIEEL imaging with Glalacton-plus. The cells were stably transfected with a LacZ construct for bacterial β-galactosidase expression and Galacton-plus activation. The total luminescence emission was imaged without an emission filter (open), and then scanned from 500 to 680 nm. Fluorescence imaging was also performed to determine the input levels of dyes. The DiD + DiR double-stained cells contained half fluorescence intensity for each dye in comparison with the corresponding single-stained cells. **c** Quantitative representation of the luminescence emission profiles as in **b**

To further test if the resonance mechanism plays a role in the intermolecular CIEEL energy transfer, we took advantage of three closely related long-chain dialkylcarbocyanines, DiI [DiIC_18_(3)], DiD [DiIC_18_(5)] and DiR [DiIC_18_(7)], as the fluorescent recipients (Fig. 3a) [17]. These lipophilic fluorophores have similar structures and only show strong fluorescence when bound to plasma membrane [17]. Depending on the number of ethelenyl (−HC = CH−) groups in the linker between the two carbocyanines, their emission fluorescent spectra range from visible yellow-green to the NIR region (Fig. 3a). DiI has the shortest 3-carbon linker and a yellow-green spectrum (λ_ex_/λ_em_ = 549/565 nm). DiD has a 5-carbon linker and a red fluorescent spectrum (λ_ex_/λ_em_ = 644/663 nm). DiR has the longest 7-carbon linker and a NIR spectrum (λ_ex_/λ_em_ = 748/780 nm). Of note, among all three dialkylcarboncyanines, only DiI has an excitation spectrum overlapping with the blue emission spectrum of Galacton-plus. DiD and DiR have no appreciable spectral overlap for resonance energy transfer. Thus, if the resonance-based mechanism is the major contributor to the observed energy transfer light production, we would expect DiI to demonstrate the highest energy transfer efficiency and DiR should be the least efficient.

To test this hypothesis, we used intact HCT116/LacZ human colon cancer cells that stably express bacterial β-galactosidase (LacZ) for Galacton-plus activation. The cells were stained with single (DiI, DiD, or DiR) or double (DiD + DiR) dialkylcarboncyanines prior to adding Galacton-plus for light production (Fig. 3b, no filter). The DiD + DiR double-stained cells contained half fluorescence strength for each dye in comparison with the corresponding single-stained ones. We then performed step-wise sequential acquiring from 500 to 840 nm to determine the emission profiles (20 nm/step, Fig. 3b, right panels). Unstained control cells showed no luminescent emission (no filter), indicating that most of enzyme-activated intermediates were subsequently quenched in the absence of any recipient dye. In contrast, cells stained with either DiD, DiR or both demonstrated dye-specific energy transfer signals. Quantitative presentation of the luminescent CIEEL signals showed great accordance with the theoretical emission spectra of the DiD and DiR (Fig. 3c). In particular, the DiR-stained cells showed the highest level of energy transfer luminescence (~200 times higher than DiI), followed by the DiD-stained cells which showed intermediate energy transfer (~10 times higher that DiI). The lower DiD energy transfer signals were not likely due to the difference in fluorescent extinction coefficient (ε_fluoro_) or quantum yield (Φ). In fact, both DiD and DiR have comparable ε_fluoro_ × Φ values. Furthermore, we observed very low levels of energy transfer to the DiI-stained cells. Although DiI has the lowest ε_fluoro_ × Φ value compared with DiD and DiR (55 % reduction), these low values do not seem to account for the more drastic reduction we observed in it’s energy transfer capability (~200 fold less than DiR). These observations are in contrast with the prediction based on resonance energy transfer mechanism, and therefore suggest that the energy transfer is not predominantly a resonance-based energy transfer mechanism. It has been found that the CIEEL intensity (corrected for quantum yield) decreases as the singlet excitation energy and the oxidation potential increase [18]. In addition, we speculate that the molecular size of fluorophore structures may also be an important factor since it positively influences the incidence of intermolecular collision.

**Fig. 4:**
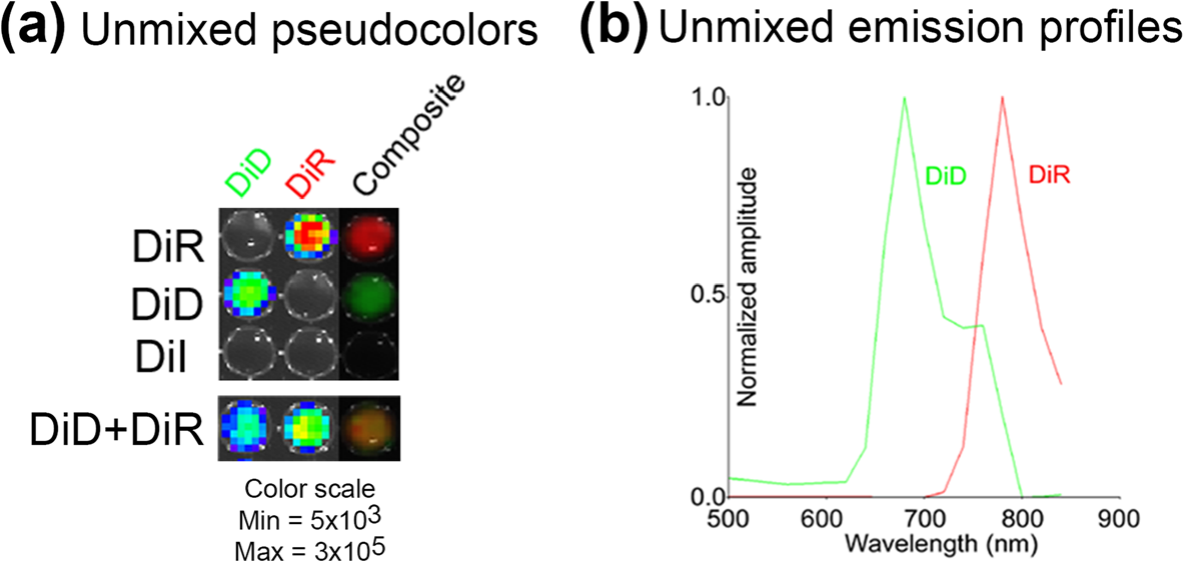
Spectral unmixing of the CIEEL signals from different fluorophores. **a** The emission profile data in Fig. 3b were spectrally unmixed using the Living Image software. A pseudocolor composite was generated where the red color indicated the relative DiR CIEEL signals and the green color indicated the relative DiD CIEEL signals. **b** The unmixed emission profiles of DiD and DiR CIEEL signals

By analyzing the emission profile data using the spectral unmixing function of the Living Image software of the IVIS imaging system, we identified two major emission components (Fig. 4). A composite image was generated to illustrate the sources of CIEEL signals (Fig. 4a, composite), where the green indicated the DiD emission and the red indicated the DiR emission. The two emission spectra fit nicely with the corresponding emission profiles of DiD and DiR (Fig. 4b). Interestingly, spectral unmixing makes it possible to perform multi-color energy transfer imaging on the same cells stained with multiple fluorescent dyes (DiD + DiR, Fig. 4b), a unique capability which would be useful for simultaneous investigation of multiple bio-physiological changes.

### Energy transfer imaging of endogenous phosphatase activity

**Fig. 5:**
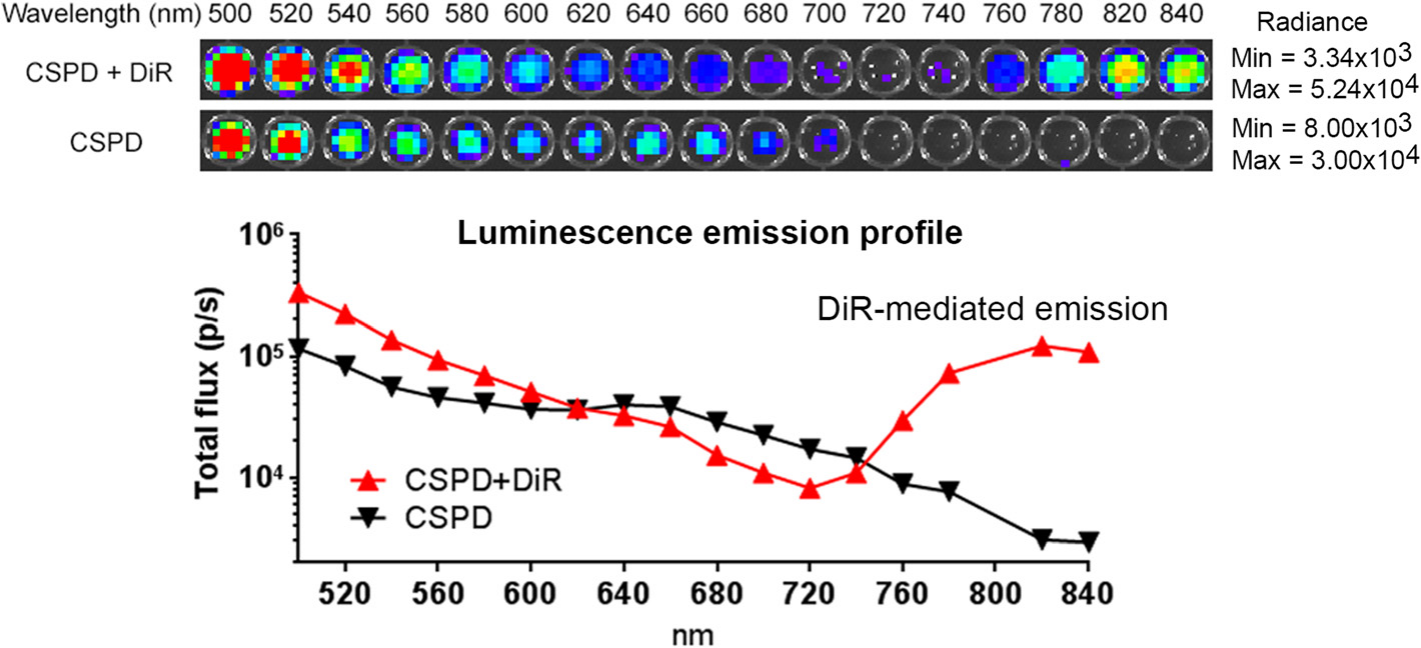
CIEEL energy transfer imaging of endogenous phosphatase activity in cells. Human MM1S myeloma cells were stained with DiR prior to CIEEL imaging with CSPD, a chemiluminescent 1,2-dioxetane substrate for alkaline phosphatase detection. Unstained cells were used as negative control. Luminescence emission levels were determined by sequential scanning from 500 to 840 nm. Quantitative representation of the imaging data showed DiR-mediated energy transfer signals in the 720–840 nm NIR region, a finding consistent with the DiR’s fluorescent emission profile

Since phosphatase activities are commonly present in many types of mammalian cells, we tested if such endogenous enzyme activities can be used for substrate activation. Structurally similar to Galacton-plus, CSPD has been widely used as a chemiluminescent substrate for detecting alkaline phosphatase activity in a variety of biological assays (Fig. 1). Hydrolytic phosphatase activity removes the protective phosphate group of CSPD for substrate activation. The substrate then generates a high-energy 1,2-dioxitane intermediate. In order to demonstrate energy capture in cells, we used DiR-stained MM1S human myeloma cells (Fig. 5). Myeloma cells are derived from B lymphoid lineage which is known to express alkaline phosphatase in the plasma membrane [19]. We observed that the endogenous phosphatase triggered CSPD activation and resulted in subsequent energy transfer to DiR. In contrast, without the recipient fluorophore, the unstained control MM1S cells only showed self-decomposition luminescence. These results were consistent with our previous findings where the Galacton-plus was used as the energy source for β-galactosidase imaging (Fig. 3).

**Fig. 6:**
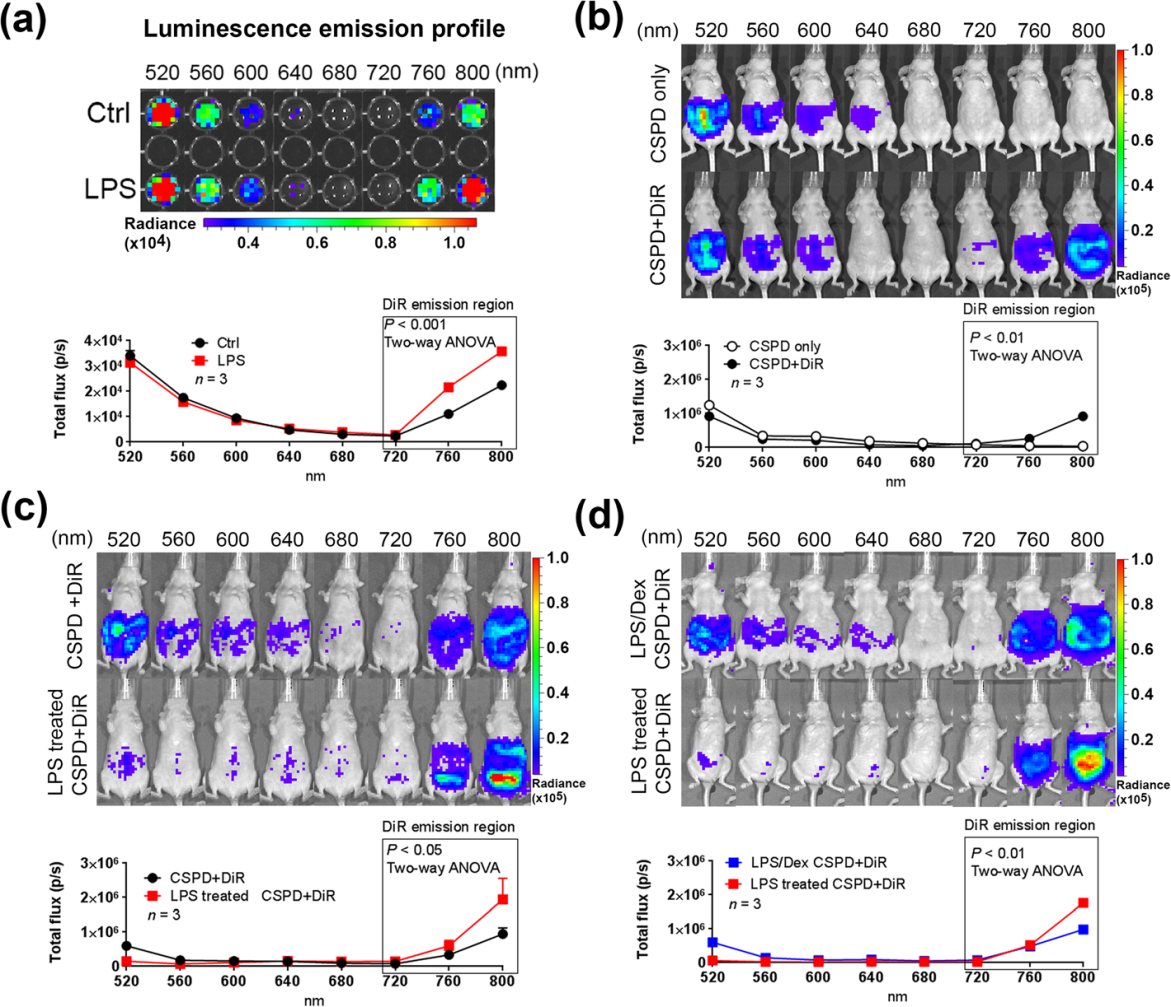
CIEEL energy transfer imaging of elevated alkaline phosphatase activity during tissue inflammation. **a** Splenocytes were harvested and cultured in the presence of LPS, a potent stimulant for inflammatory responses. The cells were then stained with DiR and subjected to CIEEL imaging with Galacton-plus. Untreated cells were used as negative control. The cells were scanned from 520–800 nm to establish their luminescence emission profiles. **b** CIEEL imaging of endogenous phosphatase activity in the peritoneal cavity of normal mice. Animals received i.p. injection of CSPD in the presence or absence of DiR, and were then subject to sequential luminescence acquisition from 520 to 800 nm. **c** NCr nude mice were i.p. treated with LPS to induce inflammatory responses in the peritoneal cavity. CIEEL imaging was performed 24 h later. Untreated control mice were used as imaging control. **d** Mice were pretreated with dexamethasone, a potent anti-inflammatory drug, and then challenged by i.p. LPS injection. CSPD-DiR CIEEL imaging was performed 24 h after LPS challenge. Error bar = SEM

We then determined if this energy transfer strategy can be used for imaging endogenous leukocyte alkaline phosphatase (LAP) activity in mouse splenocytes [20]. In this set of studies, we used a potent stimulant lipopolysaccharide (LPS) to upregulate LAP activity in splenocytes (Fig. 6a). LPS is an important component of bacterial membrane and is known for its capability to upregulate LAP activity in neutrophils [20]. Splenocytes were cultured in the presence of LPS to stimulate intracellular LAP activity. Next day, we used DiR to stain the cell membrane and then added CSPD to provide chemical energy for imaging. LPS treatment significantly increased the CSPD-DiR energy transfer luminescence in the range of 720–800 nm (Fig. 6a).

We then tested the *in vivo* application of the CSPD-DiR energy transfer for visualizing tissue inflammation in living animals. The goal of this set of studies was to use CSPD-DiR to visualize membrane-associated alkaline phosphatase (ALP) activity in inflamed tissue. This strategy relies on the membrane targeting capability of DiR to capture CSPD activated by the membrane-bound ALP during inflammation. In addition, we chose DiR over DiD for this particular application since the redder DiR CIEEL emission is attenuated less by passage through tissue, and is less subject to feed-associated imaging artifact (the alfalfa content in common food pellets strongly absorbs light in DiD’s 640–700 nm fluorescence range). Fig. 6b demonstrates endogenous phosphatase activity in the peritoneal cavity in the absence of LPS. Without the use of DiR as the energy recipient on cell membranes, the endogenous phosphatase activity produces a luminescence emission profile exclusively in the blue ~500 nm region. Of note, the blue luminescence signals could be composed of 1) the freely diffused, non-membrane bound ALP in the peritoneal fluid, or 2) membrane-associated ALP that can be subject to upregulation in response to LPS challenge and tissue inflammation. The use of DiR helps distinguish these two components as the NIR 720–800 nm emission represents the specific contribution of membrane-associated ALP.

In another study, nude mice received intraperitoneal (i.p.) injection of LPS in order to trigger inflammatory leukocytes influx into the peritoneal cavity. The day after LPS treatment, animals were i.p. injected with a mixture of CSPD and DiR for energy transfer imaging. Compared with the untreated control mice, we observed higher CSPD-DiR energy transfer luminescence in the peritoneal cavity of mice treated with LPS (Fig. 6c). Importantly, the CSPD-DiR luminescence is specific to tissue inflammation, since pretreatment of the animals with dexamethasone, a potent anti-inflammatory drug, prevent upregulation of membrane-associated ALP activity induced by LPS (Fig. 6d).

## Discussion

In this study, we demonstrated intermolecular CIEEL energy transfer and provide proof-of-principle for CIEEL-mediated imaging of enzyme activity in living animals. This strategy is a marriage of both luminescence and fluorescence imaging. By using enzyme-specific chemiluminescent substrates, CIEEL imaging has low levels of background noise. But unlike luciferase-based bioluminescence, this hybrid imaging method takes advantage of endogenous enzymes and thus eliminates the need for genetic modification and ectopic luciferase expression. As with other energy transfer imaging methods such as FRET and BRET, efficient CIEEL energy transfer requires close proximity between trigger enzymes (energy source) and fluorescent recipients (signal read-out) (Fig. 2). Thus CIEEL can also be coupled with targeting biologics to provide spatial information.

Aside from the proximity requirement, the energy transfer mechanisms of CIEEL and BRET are fundamentally different. CIEEL energy transfer is not based on resonance but requires direct molecular collision with the fluorescent recipient [11, 15]. During the collision event, the high-energy 1,2-dioxetane intermediate forms a charge-transfer complex and directly transfer its chemical energy to the fluorophore for subsequent light emission [11, 15]. Thus this feature could bypass the spectral requirement of BRET, and provides a broader choice for fluorescent signal read-outs. In a sense, this process is similar to TCPO-based glow sticks, where the activated peroxalate form a charge-transfer complex with the fluorophore to commence direct energy transfer [11, 15].

In a typical glow stick, the CIEEL mechanism begins with oxidation of the energy source, peroxalate substrate TCPO, to form a metastable 1,2-dioxetanedione, which then forms a charge-transfer complex with the recipient fluorophore. Within this transient complex, it is believe that the energy transfer begins with a single electron transfer from the fluorophore to 1,2-dioxetannedione. This extra electron in 1,2-dioxetanedione triggers its decomposition and elevation of the electron to a higher energy level. The electron is then transferred back to its original fluorophore and renders the dye in an excited state for subsequent light emission. The nature of this mechanism makes it possible to produce glow sticks with a great variety of colors, including the use of far-red and NIR fluorophores for better tissue penetration.

In this study we used enzyme-specific 1,2-dioxetane substrates as the energy source. Taking advantage of the spectral unmixing function of IVIS imaging system we are able to distinguish different CIEEL signals originated from multiple fluorophores. More importantly, this imaging method could enable us to simultaneously monitor multiple fluorophores by using a chemiluminescent substrate as the sole energy source. However, the CIEEL light production efficiency is subject to the photochemical properties of the dye. Besides the quantum yield, CIEEL efficiency could be affected by the singlet excitation energy and the oxidation potential of the fluorescent molecule. It has been found that the CIEEL intensity (corrected for quantum yield) decreases as the singlet excitation energy increases [18]. In addition, there is also a linear relationship between the CIEEL intensity (corrected quantum yield) and the oxidation potential of the molecule [18]. These factors may account for the observed difference among DiI, DiD, and DiR dyes. As DiI is the bluest dialkylcarboncyanine dye with the highest singlet excitation energy, it is the less efficient recipient dye for CIEEL luminescence.

Luciferase-based BRET still requires genetic engineering to introduce exogenous luciferase as the energy donor. Since CIEEL imaging directly takes advantage of endogenous enzymes, there is no need to exogenously introduce luciferase, either in the form of functional proteins or gene expression constructs. In this and our previous studies, we have shown the use of CIEEL imaging to visualize endogenous enzyme activities and ROS production in living animals [7]. A nano-particle system using polymerized peroxalate (since TCPO is rather unstable in aqueous environments), has been developed to monitor extracellular hydrogen peroxide and other reactive oxygen species *in vivo* [21].

In this study we also demonstrated the use of CIEEL imaging to monitor increased alkaline phosphatase activity associated with tissue inflammation. In humans, alkaline phosphatase is present in all tissues throughout the entire body. Mammalian alkaline phosphatases (ALP) is associated with microsomal and plasma membranes in many tissues. It is anchored to cell membranes by glycophosphatidylinositol (GPI) proteins. Cleavage of these proteins releases ALP from the membranes and results in elevated ALP levels in serum. The membrane-bound feature enhances CIEEL imaging when using membrane-bound DiR dye. In the peritoneal cavity, there are three types of alkaline phosphatase (ALP): 1. Free circulating alkaline phosphatase in the peritoneal fluid (ALPL, mostly released from the liver, bone or kidney) [22]; 2. Intestinal alkaline phosphatase (ALPI) associated with brush border and basal lateral plasma membrane of intestinal epithelium [23]; and 3. Leukocyte alkaline phosphatase (LAP) an isoform of ALPL found on the plasma membrane of white blood cells [24]. Inflammation induced by tissue damage and bacterial infection upregulate ALP activity in the intestines, especially in intestine cells [25–27] and infiltrating neutrophil granulocytes [24]. In this study, intraperitoneal delivery of membrane-targeting DiR and the 1,2-dioxetane ALP substrate CSPD enabled detection of increased ALP activity in inflamed gut tissues. The free circulating ALP in the peritoneal fluid should be a lesser contributor to the CIEEL energy transfer signals. On the other hand, membrane-bound ALPI and LAP could provide better CIEEL efficiency since they are in the proximity to DiR on the plasma membrane. In particular, *ex vivo* CIEEL imaging of splenocytes confirmed elevated LAP activity in response to an inflammatory stimulus (LPS).

## Conclusions

In summary, we demonstrate a novel molecular imaging technique that has potential for monitoring a wide range of biological events. It combines the advantage of both luminescence and fluorescence imaging and has the capability to perform multi-color imaging on the same subject. Chemiluminescent substrates have recently gained attention in biomedical optical imaging. Recent successes using polymeric peroxalate nanoparticlels to detect hydrogen peroxide production [21], and using luminol to detect myeloperoxidase activities in activated phagocytes [5, 12, 28] have motivated us to explore new means for luminescent imaging other than conventional luciferase-based luminescence. We believe this hybrid imaging technique will join this new trend and have the potential to become an important research tool with applications across a broad spectrum of biomedical research.

## Abbreviations

CIEEL: chemically initiated electron exchange luminescence
NIR: near-infrared
BLI: bioluminescence imaging
ROS: reactive oxygen species
S/N: signal-to-noise
MCLA: 6-(4-methoxyphenyl)-2-methyl-3,7-dihydroimidazo[1,2-a]pyrazin-3-one hydrochloride
TCPO: bis-2,4,6-(trichlorophenyl)oxalate
BRET: bioluminescence resonance energy transfer
CRET: chemiluminescence resonance energy transfer
FRET: Förster resonance energy transfer
LPS: lipopolysaccharide
ALP: alkaline phosphatase
